# Redesigning the *Aspergillus nidulans* xylanase regulatory pathway to enhance cellulase production with xylose as the carbon and inducer source

**DOI:** 10.1186/s12934-019-1243-5

**Published:** 2019-11-07

**Authors:** Patrick Ballmann, Jorge Lightfoot, Michael Müller, Stephan Dröge, Rolf Prade

**Affiliations:** 1 Prüf- und Forschungsinstitut Pirmasens e.V., Marie-Curie-Strasse 19, 66953 Pirmasens, Germany; 2 Department of Microbiology & Molecular Genetics, Oklahoma State University, Stillwater, OK 74078 USA

**Keywords:** *Aspergillus nidulans*, Biomass degradation, Biomass pretreatment, C5-sugar liquors, Cellulose hydrolysis, Cellulases, Cellobiohydrolases, Endoglucanases, Glucosidases, Enzyme production, Fungal cell factories, Xylose induced cellulase production, Xylanases, XynC, XlnR

## Abstract

**Background:**

Biomass contains cellulose (C6-sugars), hemicellulose (C5-sugars) and lignin. Biomass ranks amongst the most abundant hydrocarbon resources on earth. However, biomass is recalcitrant to enzymatic digestion by cellulases. Physicochemical pretreatment methods make cellulose accessible but partially destroy hemicellulose, producing a C5-sugar-rich liquor. Typically, digestion of pretreated LCB is performed with commercial cellulase preparations, but C5-sugars could in principle be used for “*on site*” production of cellulases by genetically engineered microorganism, thereby reducing costs.

**Results:**

Here we report a succession of genetic interventions in *Aspergillus nidulans* that redesign the natural regulatory circuitry of cellulase genes in such a way that recombinant strains use C5-sugar liquors (xylose) to grow a vegetative tissue and simultaneously accumulate large amounts of cellulases. Overexpression of XlnR showed that under xylose-induction conditions only xylanase C was produced. XlnR overexpression strains were constructed that use the *xynCp* promoter to drive the production of cellobiohydrolases, endoglucanases and β-glucosidase. All five cellulases accumulated at high levels when grown on xylose. Production of cellulases in the presence of pretreated-biomass C5-sugar liquors was investigated, and cellulases accumulated to much higher enzyme titers than those obtained for traditional fungal cell factories with cellulase-inducing substrates.

**Conclusions:**

By replacing expensive substrates with a cheap by-product carbon source, the use of C5-sugar liquors directly derived from LCB pretreatment processes not only reduces enzyme production costs, but also lowers operational costs by eliminating the need for off-site enzyme production, purification, concentration, transport and dilution.

## Background

Lignocellulosic biomass (LCB) is the single most abundant renewable hydrocarbon resource on earth [1]. The runner-up hydrocarbon resource, which is non-renewable, is petroleum. Petroleum currently provisions the world market of starter chemicals for everything from low-cost, cents-per-gallon products (gasoline and diesel) all the way to high-end substrates such as the primers for plastics, polymers and fibers [2]. Two thirds of LCB is composed of hemicellulose (C5-sugars) and cellulose (C6-sugars), the hydrocarbon substrates for fermentation processes that produce low-cost high-volume as well as high-cost low-volume chemicals [3–5]. LCB enzymatic deconstruction mechanisms are widely dispersed across the tree of life: microorganisms, bacteria, fungi, algae, plants, and others have developed specialized sets of enzymes, such as hydrolases, oxidases and monooxygenases, all of which attack cellulose, hemicellulose and lignin [6]. The canonical enzyme set, namely cellobiohydrolase(s), endoglucanase(s) and β-glucosidase(s), completely deconstruct cellulose molecules to produce glucose as the final product [7]. However, enzymatic hydrolysis is hindered by the low accessibility (recalcitrance) of the crystalline structure of cellulose to enzymes [8–10].

To overcome this natural physical resistance of LCB to an enzyme-driven digestion process, several pretreatment technologies have been developed, focused in disrupting the intermolecular hydrogen bonds that make LCBs recalcitrant [11–13]. Pretreatments include mechanical (physical) methods, such as high-pressure homogenization [14], crushing, microwave [11], ultrasonic treatments [15] and vibrating ball mill grinding and compression techniques [16]. Chemical pretreatment technologies include Fenton oxidation chemistry-based treatments that focus on the production of hydrogen peroxide to break down recalcitrant glycoside and lignin-bonds by oxidation [17], treatments with acids [18] or alkali [19], ionic liquids or extraction with organic solvents [12]. Often times, chemical and physical methods are combined [11, 20, 21] and result in treatments such as steam explosion [20, 22], ammonia fiber expansion (AFEX) [23, 24], CO_2_ explosion [25] and SO_2_ explosion [26]. The bottom line on LCB pretreatments is that irrespective of the method, there is always partial decomposition of the hemicellulosic fraction, which contains an abundance of the C5-sugar xylose [4, 10, 27].

For large-scale production of enzymes that break down LCBs, fungi have traditionally been used as cell factories to manufacture cellulases, xylanases and other auxiliary activities [28–32]. There have been considerable efforts to increase recombinant protein yields in *Aspergilli* by transcription factor engineering [33–35], reduction of extracellular protease activity [36, 37] and identification of strong promoters and protein secretion signals [38, 39]. Filamentous fungi such as *Trichoderma* and *Aspergillus* are able to use a broad range of sugars such as hexoses (C6-sugars) and pentoses (C5-sugars) as a carbon source to promote vegetative growth, however these carbon sources are insufficient to induce the synthesis of cellulases and other LCB degrading enzymes [40–42].

While fungi have been genetically engineered to secrete economically adequate yields of enzymes, the operational costs of synthesizing them continue to be excessive, largely because they demand an expensive carbon source to cultivate the vegetative tissue necessary to synthesize client proteins. Moreover, there exist the added costs of making them on distant sites, purification, concentration, conditioning and delivery to biomass processing sites [43–46].

**Fig. 1:**
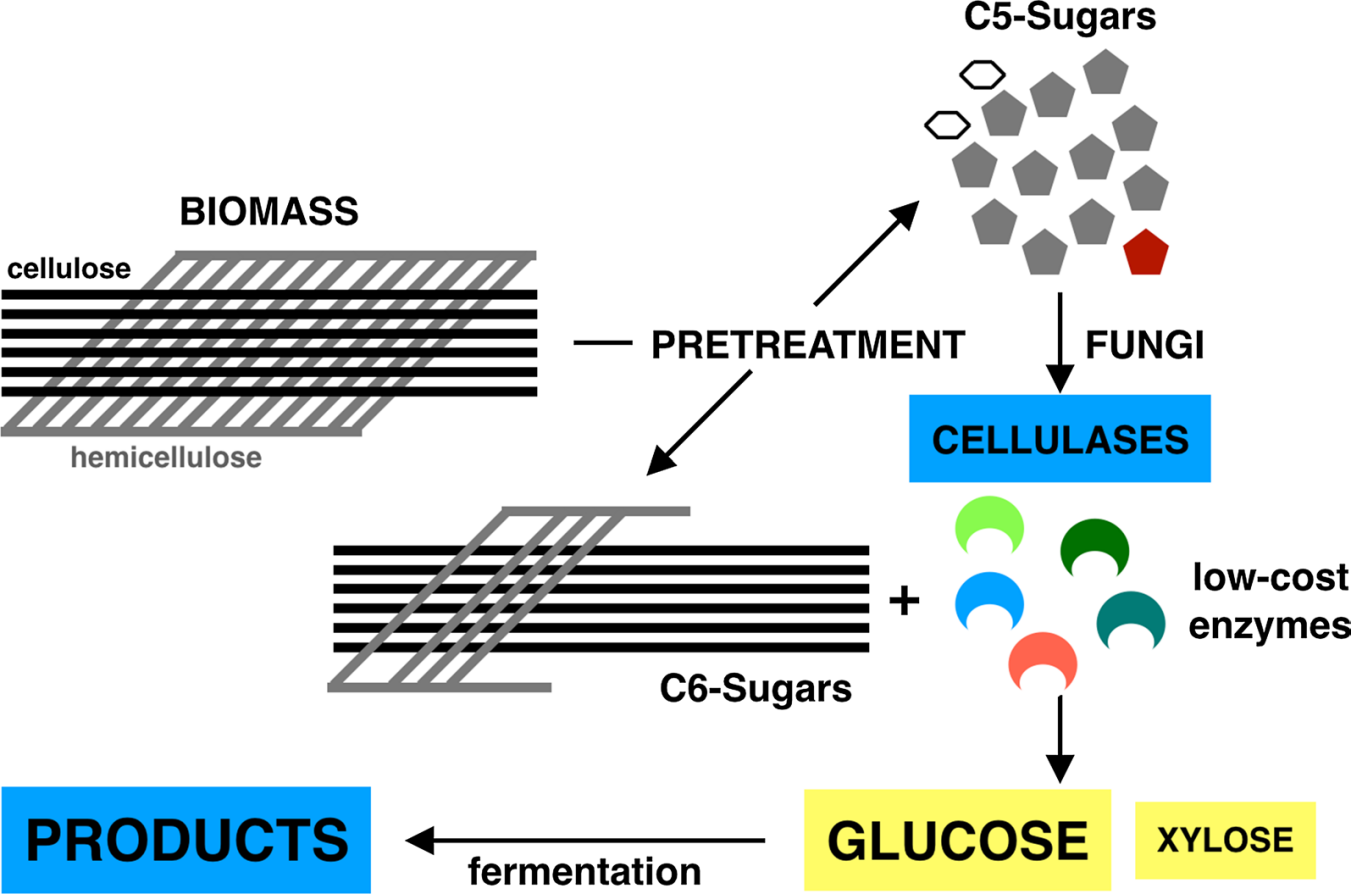
Schematic of total on-site biomass degradation with low-cost enzyme production. BIOMASS main hydrocarbon components are cellulose and hemicellulose (both account for about ~ 60% of the total dry weight of biomass [22]. PRETREATMENT technologies make cellulose (C6-sugars) accessible to enzymatic hydrolysis but compromise the integrity of hemicellulose, rendering C5-sugars which could be used to make low-cost enzymes that degrade cellulose (C6-sugars) generating GLUCOSE that is converted into fermentation PRODUCTS

Xylose found in pentosan-containing pretreated biomass liquors (PPTB), the byproducts of LCB pretreatments, is a cheap alternative carbon source that can be used as a substrate to manufacture enzymes. Using PPTBs as the raw material for the production of cellulases with fungal cell factories opens the prospect for low-cost enzyme production (Fig. 1). The problem with low-cost on-site enzyme production is that while most native fungi grow well with the by-product xylose as a carbon source, they are unable to synthesize large quantities of cellulases in the presence of PPTBs [47].

Fungi synthesize multiple forms of cellulases such as cellobiohydrolases, endoglucanases and ß-glucosidases [48–51] only if induced with C6 sugar derivatives [52], cellulose, cellobiose, or trans glycosylated cellobiose products such as sophorose [52, 53]. Native fungi growing on C5-sugars (xylose) are unable of synthesize cellulases. Fungi synthesize multiple forms of hemicellulases such as xylanases, xylosidases, mannanases, arabinofuranosidases, arabinases and xyloglucanases only if induced with C5-sugar derivatives such as xylan, xylo-oligomers, xylobiose or xylose [54]. Induction of hemicellulases is mainly regulated by the positive transcription factor activator XlnRA [55]. Thus, if one wants to produce large quantities of cellulases by using C5-sugars one has to change the way fungi activate the expression of cellulases by manipulating the activating transcription factors and the promoter that drives cellulase expression [53, 56]. The research reported here resolves this problem by redesigning the *Aspergillus nidulans* native cellulase gene regulatory circuit, switching the induction mechanism from cellulose to xylose. The strains constructed in this study grow well in xylose, simultaneously producing and secreting large amounts of cellulases. We tested the production of two cellobiohydrolases, two endoglucanases and one ß-glucosidase.

Replacing expensive substrates with a cheap by-product carbon source, PPTB directly derived from LCB pretreatment processes, not only reduces enzyme production costs, but also lowers operational costs, such as off-site enzyme production, purification, concentration, transport and dilution [43–46].

## Results and discussion

In this work, we aimed to switch *A. nidulans* from its natural transcriptional induction regulatory mechanism driven by cellulose signals to a xylose-driven induction mechanism, thus allowing *A. nidulans* to grow on xylose and simultaneously be induced by that same C5-sugar to produce large amounts of cellulases.

To determine which xylanase promoter would most strongly induce cellulase production in the presence of xylose, we replaced 1 kb of the upstream *cbhC* (cellobiohydrolase C, AN0494) promoter region with ~ 1 kb of four xylanase promoter regions, namely *xynAp* (xylanase A, AN3613), *xynBp* (xylanase B, AN9365), *xynCp* (xylanase C, AN1818) and *xynEp* (xylanase E, AN7401). In the presence of xylose, *xynCp* showed the best performance in secreting cellobiohydrolase (data not shown). Even though all tested promoters secreted cellobiohydrolase (*cbhC*) at higher levels than wild-type, the total amount of cellobiohydrolase observed in the medium was less than expected, and some of the promoters were affected by pH and strong carbon catabolite repression (data not shown).

### XlnR overexpression and xylose induction

**Fig. 2:**
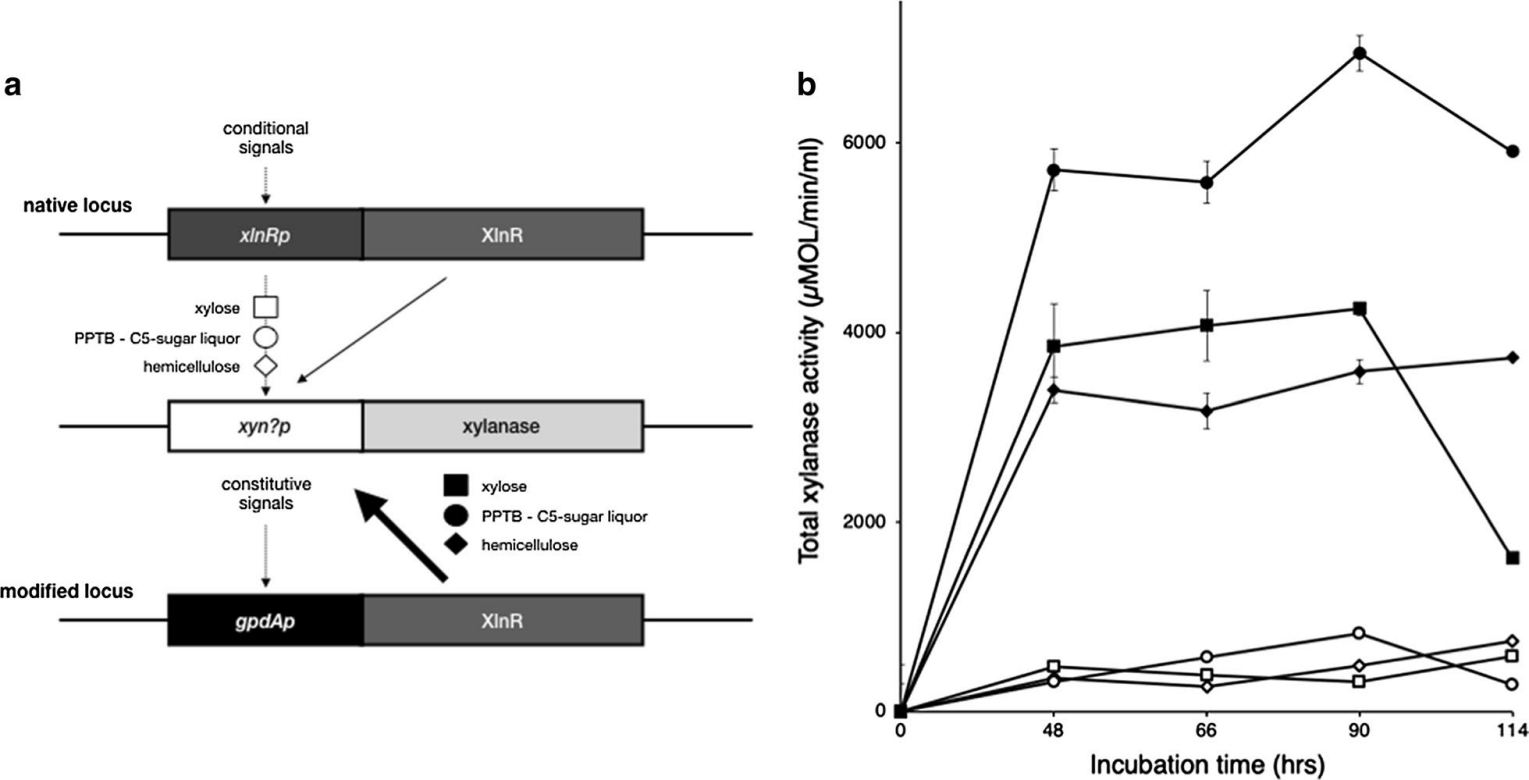
Constitutive overexpression of XlnR superinduces xylanase production. **a** Genetic description of promoter modifications on the XlnR locus which result in the overexpression of XlnR and induction of xylanases by C5-sugar signals. Open and closed symbols denote inducers, xylose (squares), PPTB (circles) or hemicellulose (diamonds) used to produce xylanase by WT (A773) and PFIX7, respectively. **b** Xylanase activity in PFIX7, XlnR over-expression driven by the *gpdAp* promoter (closed symbols) and WT (A773) parent strain (open symbols) grown with 1% xylose (squares), 1% hemicellulose (diamonds) or 1% PPTB (circles)

We thus decided to enhance the expression of client proteins driven by xylose promoters by constructing a *xlnR* (xylanase transcription activator) constitutive overexpression strain. *xlnR* was placed under the control of the *gpdAp* promoter, which drives constitutive and strong expression of G3P dehydrogenase (Fig. 2a). For a detailed description of the DNA fragment fusion construction strategy, genomic data and genetic validation of genetic modifications, refer to the Additional file 1.

Figure 2 compares xylanase production of PFIX7, the *gpdAp*::*xlnR* overexpression strain, with the WT (A773) when growing in media containing 1% xylose, 1% hemicellulose or PPTB (2% xylose, 0.37% arabinose and 0.28% glucose). The vegetative growth rates of PFIX7 were comparable to WT (A773) (data not shown) in all C5-sugar sources, but PFIX7 secreted large amounts of xylanases while growing on C5-sugar substrates such as xylose (squares), PPTB (circles) and hemicellulose (diamonds).

Table 1 shows xylanase production in WT (A773) and PFIX7 growing on 2, 4 or 6% xylose. With 2% xylose, the WT (A773) accumulated 505 ± 70 U (µmol/µl/min) whereas PFIX7 produced 14,023 ± 4329 U (µmol/µl/min), representing a 26-fold increase in xylanase accumulation. With 4% or 6% xylose, the overaccumulation of xylanase did not increase further. Tamayo-Ramos observed a 200-fold increase in enzyme activity (RhaA) of *A. nidulans* strains overexpressing XlnR (*gpdAp*::*xlnR*) and growing on hemicellulose by measuring the reporter α-l-rhamnosidase (RhaA) on strains where the *xynAp* and *xynBp* promoters were fused to *rhaA* [35].

**Table 1:** Xylanase overexpression and enhanced extracellular protein secretion in PFIX7

	Inducer	Xylanase	Total protein	CbhC
	Xylose (%)	U (µmol/µl/min)^a^	µg/ml	U (pNPC)^b^
WT	2	505 ± 70	259 ± 78	0.63 ± 0.29
	4	628 ± 85	260 ± 39	0.36 ± 0.06
	6	593 ± 32	302 ± 32	0.45 ± 0.03
PFIX7	2	14,023 ± 4329	1442 ± 349	4.23 ± 0.06
	4	16,248 ± 3091	1176 ± 233	4.01 ± 0.02
	6	14,958 ± 2746	1225 ± 211	2.59 ± 0.01

^a^Substrate was beechwood xylan; ^b^ substrate was pNPC (*p*-nitrophenyl β-d-cellobioside)

We measured cellobiohydrolase (CbhC) activity as a control, as CbhC is not to be under the control of XlnR but under the control of cellulose signals, although it has been reported that in some fungi cellulases are also regulated by XlnR [57, 58]. Table 1 shows that PFI-X7 CbhC had a 7 (0.63 to 4.23 U), 11 (0.36 to 4.01 U)- and 6 (0.45 to 2.59 U)-fold increase in cellobiohydrolase activity in 2, 4 and 6% xylose respectively. Tamayo-Ramos [35] observed that the total amount of protein secretion was enhanced in XlnR over-expressing strains. Therefore, we also measured the total amount of protein secreted, and observed that PFIX7 displayed a 4- to 6-fold increase in total protein secretion (Table 1). The observed protein secretion augmentation was consistent with the increased CbhC activity. Thus, the enhanced CbhC secretion observed in PFIX7 is most likely the result of the improved protein secretion activity driven by XlnR, rather than the specific regulation of cellulase promoters by XlnR. These results corroborate the finding by [35, 57, 58].

From the data shown in Fig. 2 and Table 1 it seems fair to suggest that XlnR strongly regulates the expression of xylanase activity, while leaving open the possibility that it regulates other activities, such as auxiliary hemicellulases and perhaps cellulases. Moreover, from Fig. 2 and Table 1 it remains unclear whether XlnR regulates the expression of only one, two or all five xylanases (*xynA*, *xynB*, *xynC*, *xynD*, and *xynE*) encoded by the *A. nidulans* genome [7].

**Fig. 3:**
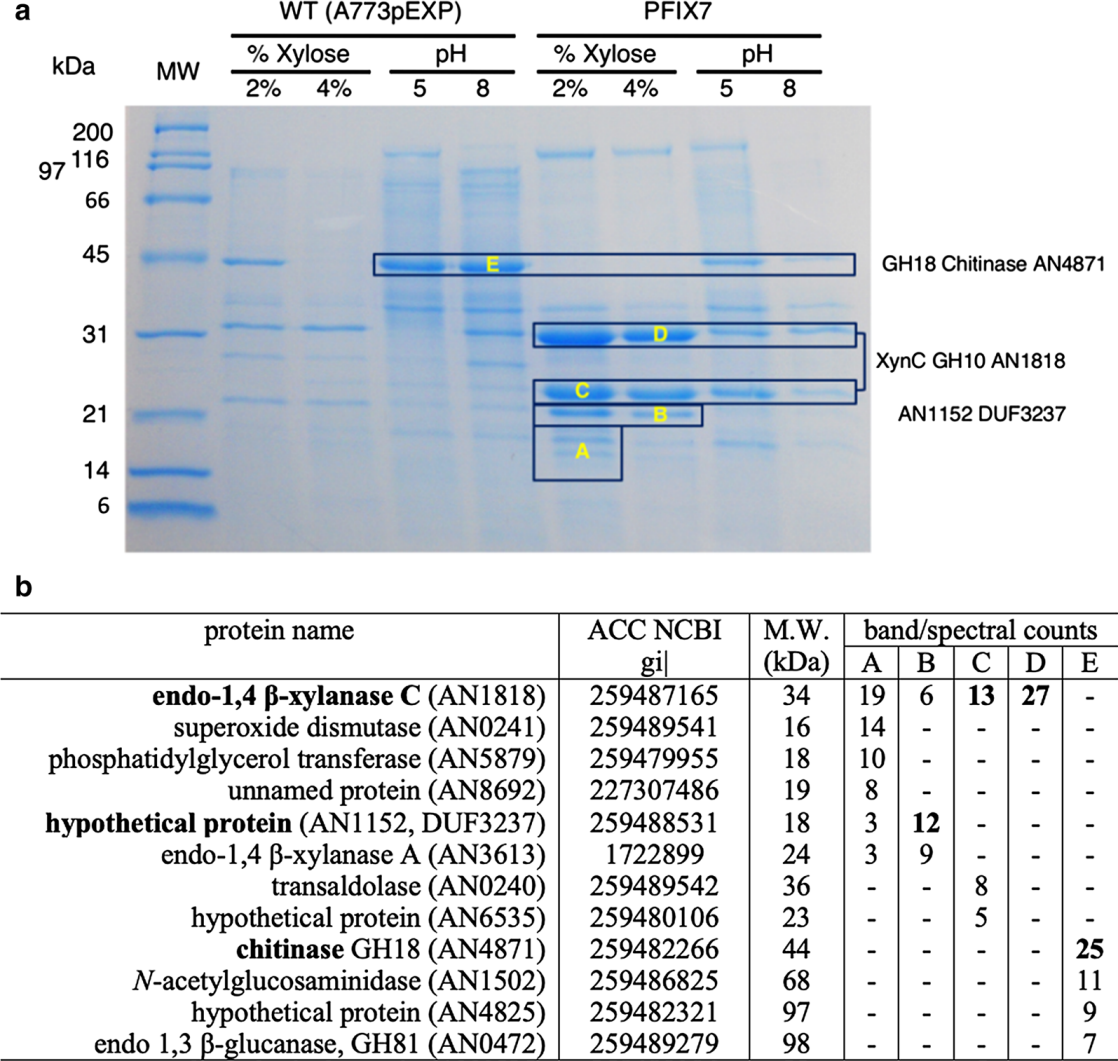
Endo-1,4 β-xylanase C (XynC) is the major xylanase secreted by PFIX7, the XlnR overexpression strain, when grown on xylose. **a** SDS-PAGE showing total secreted proteins in WT (A773) and PFIX7 when growing with 2 or 4% xylose at pH 5 or pH 8. Boxes indicate major proteins present under various conditions, and bands identified by letters were excised and analyzed by LC/MS–MS. **b** Table correlating protein IDs (protein name) with LC/MS–MS spectral counts (abundance) of excised protein bands indicated in **a**

Thus, we decided to determine which enzymes were most strongly secreted by PFIX7 when stimulated with xylose. Figure 3a shows protein profiles (SDS-PAGE) of enzymes secreted by WT (A773) and PFIX7 growing on xylose. Figure 3b lists the spectral counts, determined by LC–MS/MS, of overexpressed protein bands A, B, C, D and E. Remarkably only three proteins were over-secreted in PFIX7 when cultivated on xylose: a chitinase (GH18, band E), xylanase C (bands C and D), and a protein of unknown function AN1152 (band B). Only small amounts of xylanase A, and no other xylanases (B, D or E) were detected by LC–MS/MS (Fig. 3b). In our experiment, which only examined hyper-secreted proteins of *A. nidulans* grown on xylose as the sole carbon source, the XlnR-induced and secreted xylanase (PFIX7) comprised two versions of xylanase C, namely a full-length version (~ 34 kDa, band D with CBM1) and a truncated version with a catalytic domain and no CBM1 domain (~ 22 kDa, band C).

Taking into consideration all of our findings for the overexpression of XlnR in media growing on C5-sugars (Figs. 2, 3a, b, Table 1), we conclude that overexpressing XlnR (PFIX7) results in predominant secretion of xylanase C (XynC) when mycelia are grown on xylose. Thus, using the *xynCp* promoter to drive the production of client proteins (cellulases) in a strain that overexpresses XlnR is likely to accumulate large amounts of client proteins.

### Xylose-induced production of cellulases

To test the assumption that XlnR overexpression would drive accumulation of potential client proteins driven by the *xynCp* promoter, we constructed a series of strains that overproduce five model cellulase genes that are predicted to be necessary to completely convert a cellulose molecule into glucose. Based on the evidence reported by Segato and cols. ([7] and others cited therein), the selected model genes included two cellobiohydrolases (CbhB and CbhC), two endoglucanases (EglA and EglB) and one β-glucosidase (BglA). Plasmids bearing *xynCp*::CP (client protein) constructs were transformed into PFIX7, and transformants were selected based on the amount of secreted client protein (CP).

**Fig. 4:**
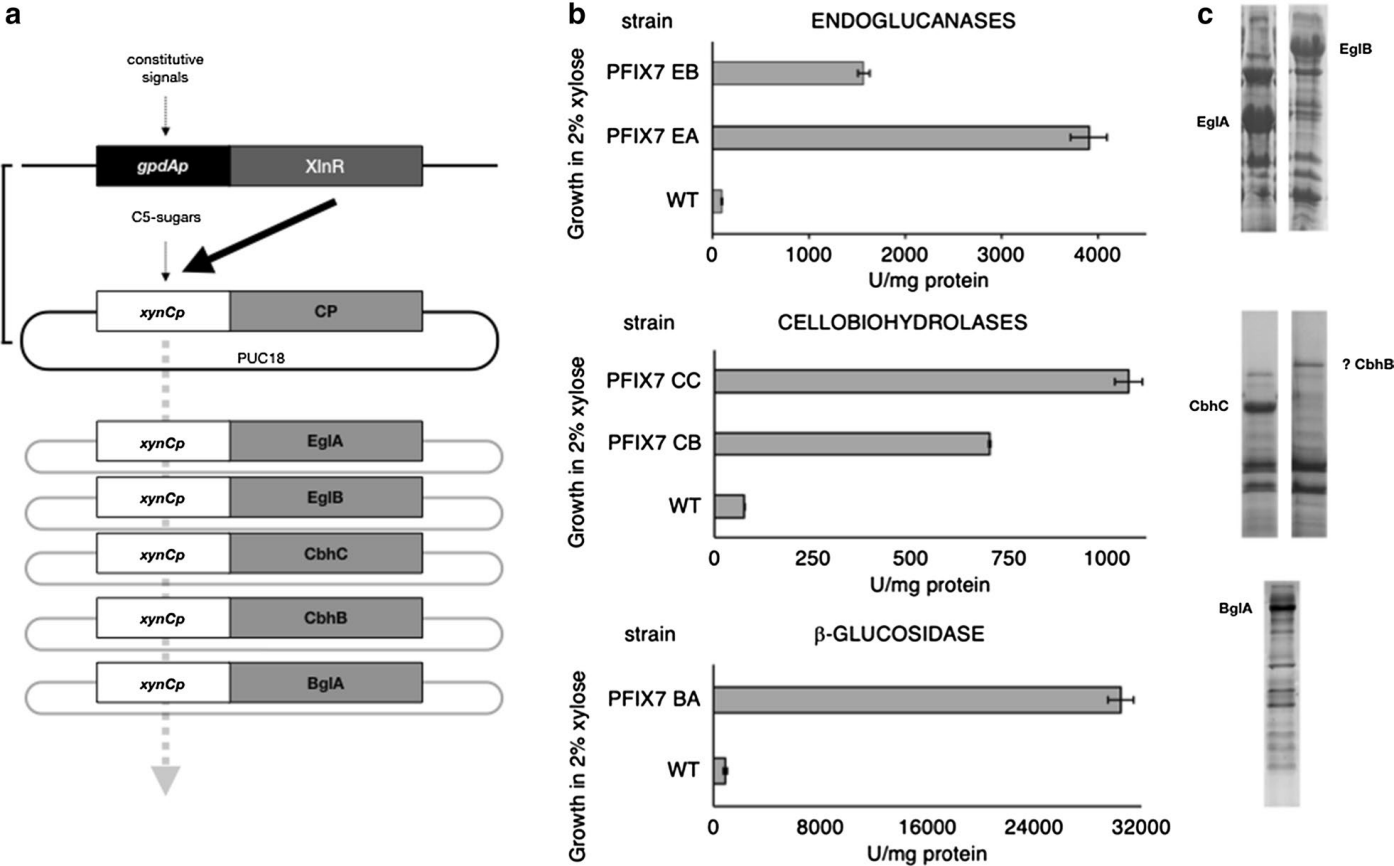
*xynCp*-driven client protein hyperaccumulation induced by xylose in strains overexpressing XlnR. **a** Plasmids carrying the *pUC18*_*UP*_::*pyroA*::*xynCp*::CP_ORF_::*pUC18*_*DW*N_ GAT construct were transformed into PFIX7 (XlnR-overexpressing) strain, and recombinants were selected based on client protein production rates. Specific enzyme activity (**b**) and protein accumulation verified by SDS-PAGE (**c**) of five client proteins, two endoglucanases (strain PFIX7-EA, enzyme EglA, strain PFIX7-EB, enzyme EglB), two cellobiohydrolases (strain PFIX7-CC, enzyme CbhC, strain PFIX7-CB, enzyme CbhB) and a β-glucosidase (strain PFIX7-BA, enzyme BglA)

Figure 4 shows total enzyme activity and protein accumulation of five model genes (CPs) grown in the presence of 2% xylose. For the endoglucanases EglA and EglB, we found 3908 ± 190 and 1570 ± 60 enzyme units per milligram total protein, respectively (Fig. 4b). For the cellobiohydrolases CbhB and CbhC, we found 702 ± 3 and 1054 ± 35 enzyme units per milligram total protein, respectively (Fig. 4b). For the β-glucosidase BglA, we found 30,436 ± 964 enzyme units per milligram protein (Fig. 4b). SDS-PAGE of crude unfiltered extracts (Fig. 4c) showed that all of the enzymes overaccumulated in the medium. For CbhB, we could not unambiguously detect a clear protein band on SDS-PAGE gels despite detecting increased activity (702 U per milligram protein).

The above result is promising because the engineered strains (PFIX7-EA, PFIX7-EB, PFIX7-CB, PFIX7-CB and PFIX7-BA) accumulate large amounts of client proteins relative to the production of cellulases in the WT (A773) when grown on xylose. The engineered strains, PFIX7-EA, PFIX7-EB, PFIX7-CB, PFIX7-CB and PFIX7-BA, showed 35-, 40-, 16-, 9- and 14-fold increases in extracellular specific protein accumulation of β-glucosidase, endoglucanase A, endoglucanase B, cellobiohydrolase B and cellobiohydrolase C, respectively.

### Production of xylanases and cellulases with PPTB

Next, we examined the prospect of using PPTBs both as a C5-sugar carbon source for growth and as an inducer to produce cellulases. Because PPTBs are a byproduct of LCB pretreatments, they primarily contain xylose; however, other sugars and phenols are also present. The PPTB routinely obtained in our laboratories by treating wheat-straw (LCB) with diluted nitric acid at 160 °C for 30 min and then concentrating in a vacuum evaporator contains 162 g/l (76.7%) of xylose, 29.4 g/l (14%) of glucose, and 19.7 g/l (9.3%) of arabinose as potential carbon sources.

We tested two media formulations: a minimal medium composed of Clutterbuck salts [59] amended with xylose (30 g/l) and a PPTB medium containing Clutterbuck salts [59] amended with PPTB (adjusted to 30 g/l of xylose, thus corresponding to glucose and arabinose levels of 5.6 g/l and 4.2 g/l, respectively). Three strains were examined for overproduction of enzymes in PPTBs: PFIX7, which due to overexpression of the XlnR transcription factor naturally over-produces xylanase; PFIX7-EA, which overexpresses endoglucanase A (EglA); and PFIX7-BA, overexpressing ß-glucosidase (BglA).

Table 2 shows that all three strains, PFIX7, PFIX7-EA and PFIX7 BA, produced large amounts of target proteins in xylose-only media, with 24,324 ± 3479 U of xylanase, 3191 ± 85 U of endoglucanase and 1749 ± 93 U of ß-glucosidase produced, respectively. PPTB-containing media also strongly induced target protein production to 29,222 ± 859 U of xylanase, 4008 ± 395 U of endoglucanase and 1952 ± 133 U of ß-glucosidase, respectively.

**Table 2:** Cellulase and xylanase production in media containing C5-sugars

	Enzyme measurements		Secreted	Spent	Enzyme	
	Activity	Specific	Proteins	Sugar	Productivity	
	U/ml	U/mg prot^d^	g/l	%	U/l h	Y_P/S_
Xylose amended minimal medium						
PFI-X7	2760 ± 6^a^	4058	0.68	96	38,333	35,646
PFIX7-EA	283 ± 26^b^	340.9	0.83	96	3930	4598
PFIX7-BA	155 ± 18^c^	176,1	0.88	93	2152	2662
PPTB amended minimal medium						
PFI-X7	2473 ± 51	4666	0.53	80	34,347	49,420
PFIX7-EA	328 ± 14	385.9	0.85	86	4556	6132
PFIX7-BA	161 ± 7	206.4	0.78	83	2236	3239

^a^Xylanase activity; ^b^ endoglucanase activity; ^c^ beta-glucosidase activity, ^d^ U per mg of secreted protein

The amounts of xylanase, endoglucanase and ß-glucosidase produced in xylose-only and PPTB-amended media were similar (Table 2), indicating that the presence of other sugars in PPTB such as glucose and arabinose did not negatively affect the enzyme production process. Table 2 also shows that carbon source consumption was slightly different. In xylose-only media, consumption was almost complete, above 90%, but in PPTB-containing media, consumption was slightly reduced but still above 80%.

For comparison purposes (Table 2) we report our enzyme productivity measurements in various ways: U/ml (amount of enzyme per mL of medium), U (total amount produced in the fermentation system), U/g (of biomass (mycelium) and U/mg (of total amount of secreted protein). In Table 3 we tried to gather the best published enzyme production rates for xylanases, endoglucanases and ß-glucosidases produced in different protein expression hosts with various inducers. In PPTB, our system produced 4666 U/mg protein of xylanases. In comparison, xylanases produced by *Pichia pastoris* range between 923 and 1533 U/mg of xylanase, and xylanase production in *A. awamori* using the PFE2 expression plasmid reached 149.6 U/mg. For endoglucanases, our PPTB system produced 385.9 U/mg protein, whereas endoglucanases produced in *P. pastoris*, *Escherichia coli* or *A. nidulans* reached between 6.78 and 256 U/mg of endo enzymes. Our PPTB system produced 206.4 U/mg protein of ß-glucosidases, whereas *P. pastoris* produced 66.6 to 258 U/mg.

**Table 3:** Heterologous protein expression (and/or)/secretion of xylanases, endoglucanases and ß-glucosidases

Enzyme/class	Gene (ORF) source	Expression host/expression system	Inducer	Activity		Refs.
				#	Unit	
Xylanases						
XYN2	*T. reesei*	*P. pastoris*/PICZ	Methanol	1435	U/mg	[75]
xynC	*A. nidulans*	*P. pastoris*/PICZ	Methanol	923	U/ml	[76]
GH11	*M. thermophila*	*P. pastoris*/PICZ	Methanol	1533.7	U/mg	[77]
GH11	*A. clavatus*	A. awamori/pFE2	Maltose	149.6	U/mg	[78]
Endoglucanases						
GH6	*C. cinerea*	*P. pastoris*/PICZ	Methanol	118.75	U/mg	[79]
GH5	*T. aurantiacus*	*P. pastoris*/PICZ	Methanol	190	U/mg	[80]
GH12	*A. terreus*	*A. nidulans*/pEXPYR	Maltose	256	U/mg	[81]
ß-Glucosidases						
GH3	*M. thermophila*	*P. pastoris*/PICZ	Methanol	258.7	U/mg	[82]
GH3	*P. thermophila*	*P. pastoris*/PICZ	Methanol	66.6	U/mg	[83]
GH1	*P. thermophila*	*P. pastoris*/PICZ	Methanol	192.7	U/mg	[84]

## Conclusion

Here we report on a succession of genetic interventions in *Aspergillus nidulans* that redesign the natural regulatory circuitry of cellulase genes in such a way that recombinant strains use C5-sugar liquors (PPTB) to grow a vegetative tissue and simultaneously produce large amounts of cellulases. Five cellulases, two cellobiohydrolases (CbhB and CbhC), two endoglucanases (EglA and EglB) and a β-glucosidase (BglA) accumulate at high titers when cultivated with PPTB C5-sugars. Cellulase production rates with PPTB was comparable to other heterologous expression systems, *P. pastoris*, *E. coli* and fungal cell factories. Recouping PPTBs to streamline the biomass degradation process by integrating pretreatment technologies with the use of C5-sugars to produce the enzymes needed to digest pretreated biomass should result in significant cost reductions applied to the entire biomass degradation process. We are currently investigating the feasibility of large-scale production of cellulases with PPTBs.

## Materials and methods

### Chemicals and specialty chemicals

General chemicals, cellulosic and hemicellulosic substrates were purchased from the best source possible, Sigma Aldrich (St. Louis, MO) and Megazyme (Ireland, UK). Phosphoric acid swollen cellulose (PASC) was prepared according to [60].

Wheat straw was harvested in 2015 from a local farmer in Rhineland Palatinate (Bad Kreuznach, Germany). The composition was determined according to the method suggested by the National Renewable Energy Laboratory (NREL) for measurement of structural carbohydrates and lignin [61]. The wheat straw had 37.1% (w/w) cellulose, 22.3% (w/w) hemicellulose, 16.8% (w/w) lignin, 9% (w/w) extractives and 4.3% (w/w) ash. HPLC analytics were done with the Metacarb 87H column (300 mm × 7.8 mm) purchased from Agilent Inc. (Santa Clara, CA, USA). All used chemicals were purchased from VWR International (Radnor, PA, USA).

### Production of the xylose-containing liquefied wheat straw hydrolysate (PPTB)

The PPTB, pentosan containing pre-treated biomass liquor was prepared by diluted acid hydrolysis of wheat straw in a 100-l stainless steel reactor. The vessel was heated with direct steam injection until the desired temperature was reached. In a previous study, the optimized treatment process parameters for high xylose and low-by-product concentration were estimated [62]. Briefly, dried wheat straw (15% v/w, dry matter content) and diluted nitric acid (0.45% v/v) was heated up at 160 °C for 30 min. After the pretreatment the pentose-rich liquor was separated from the solid biomass. The pre-hydrolysate solution was concentrated in a rotary evaporator at 75 °C and 110 mbar to enhance the storability of the pre-hydrolysate. The concentrated solution contained 162 g/l xylose, 29.4 g/l glucose and, 19.7 g/l arabinose. Pretreatment by-products such as furfural and 5-HMF were removed through the evaporation process. The concentrated hydrolysate was stored at − 20 °C.

### Strain construction

Standard *A. nidulans* minimal medium (MM) and general cultivation techniques were used throughout this work and are based on the work by Guido Pontecorvo [63, 64] and John Clutterbuck [59]. All strains constructed in this work were derived from *A. nidulans* A773 (*wA3*, *pyrG89*, *pyroA4*) purchased from the Fungal Genetics Stock Center (FGSC, St. Louis, MO). All gene models and promoters were from *Aspergillus nidulans* FGSC4 (https://www.ncbi.nlm.nih.gov/assembly/GCF_000149205.2) and analyzed using the AspGD database (http://aspgd.org [65]) Primers and Gibson Assembly hybrid primers were designed using the NEB Builder Assembly Tool (http://nebuilder.neb.com).

Three types of strains were constructed in this study; First the resident CbhC (AN0494) promoter (*cbhCp*) was replaced with four xylanase promoters (*xynABCEp*) in such a way that recombinant strains induce the production of cellobiohydrolase by xylose, second a XlnR_(ORF)_ overexpression strain (PFIX7) was constructed by *pabaA* ectopic integration of a *gpdAp*::XlnR_(ORF)_ DNA fragment, and third, xylose induced client protein constructs were randomly introduced into a XlnR overexpressing strain (PFIX7). For a detailed description of DNA fragment fusion construction strategy, genomic data and genetic validation of genetic modifications refer to Additional file 1.

In all types of strain constructions, a linear hybrid recombinant DNA fragment was synthesized using Gibson Assembly Technology, GAT [66, 67] using hybrid primers, Gibson Assembly Master Mix (New England Biolabs, US) and Phusion DNA Polymerase (New England Biolabs, US). DNA fragment size and DNA sequence verified hybrid DNA fragments were transformed into A773 or PFIX7 protoplasts. In the case of promoter replacements, a single gene replacement event at the *cbh1* locus was selected for each *xyn(p)* promoter replacement by uracil/uridine sufficiency and by diagnostic PCR showing single integration (replacement) into the *cbh1* locus. For the XlnR overexpression, the hybrid DNA fragment was integrated into the *pabaA* locus by a double crossover event disrupting it. Recombinants with a single gene replacement event were searched with diagnostic PCR and the resulting strain PFIX7 tested for XlnR over-expression.

For the client protein xylose induced strains we created plasmids carrying the *pUC18*_*UP*_::*pyroA*: *xynCp*::CLIENT_ORF_::*pUC18*_*DWN*_ GAT construct that was transformed into PFIX7 (XlnR overexpressing) strain and recombinants selected based on the level of client protein production rates. Even though we did not check for multiple integration events in single transformants we screened at least 100 transformants for high secretion levels of client proteins.

### Preparation of total extracellular protein extracts

Unless otherwise stated, 5 ml of extracellular fluid (medium) harvested from mycelia grown for 24, 36 or 48 h were treated with 3 kDa cutoff Nanosep^®^ ultrafiltration Omega^™^ membrane columns (PALL Corp. USA) and washed with 500 µl of 50 mM ammonium acetate (NH_4_CH_3_CO_2_) buffer pH 5 before 10× concentration to a final volume of 50 µl.

### Protein quantification and SDS–polyacrylamide gel electrophoresis

Total protein content was measured in microtiter dishes using the Bio-Rad assay reagent (Bio-Rad Laboratories, USA), using a procedure based on the Bradford method [68, 69] with bovine serum albumin as standard. Absorption was measured using a UV–Vis 96-well plate reader (Tecan Infinite M200, Männedorf, Switzerland) at 595 nm. Quality of total extracellular protein extracts was validated for integrity by SDS polyacrylamide gel electrophoresis according to Shapiro [70].

### Liquid chromatography-tandem mass spectrometry

For LC–MS/MS analysis bands of a fully resolved SDS-PAGE gel (shown in Fig. 3a) were excised and processed for LC–MS/MS according to [71] with modifications. Isolated gel bands were reduced with Tris (2-carboxyethyl) phosphine, alkylated by 2-iodoacetamide, digested for 6–16 h with 8 μg/ml trypsin using ammonium bicarbonate buffer and analyzed by LC–MS/MS using LTQ-Orbitrap XL hybrid mass spectrometer (Thermo Scientific). The LC–MS/MS raw files were used for database Mascot (version 2.2.04, Matrix Science, London UK) searches run on a NCBI Aspergillus nidulans FGSC4 subsets. Searches were validated using Scaffold (version 4.0.7, Proteome Software Inc. Portland, OR) with a protein threshold of 5% FDR and a peptide threshold of 99%.

### Free sugar (reducing end) determinations

Free sugar determinations were used in two types of experiments: (1) to determine the activity of enzymes that use a non-reducing substrate releasing reducing products (sugars) and (2) to quantitate the amount of reducing sugar consumed. In both cases we used the dinitrosalicylic acid (DNS) assay developed by Sumner and Graham [72] for detection of reducing sugars. The DNS reducing sugar assay was based on the method described by Miller [73] and adapted to a microtiter dish scale. The DNS reagent we used contained 0.75% dinitrosalicylic acid, 0.5% phenol, 0.5% sodium metabisulfite, and 1.4% sodium hydroxide, 21% sodium and potassium tartarate.

### Determination of enzyme activities

Xylanase and endoglucanase activity were determined using beechwood hemicellulose or carboxymethylcellulose (CMC) as a substrate, respectively and activity measured by the release of reducing sugars that react with DNS [73]. Briefly to 300 µl of 1% beechwood xylan or 1% CMC, 50 mM ammonium acetate buffer 10–50 µl of total extracellular protein extract (treated as described in 2.2) was added and reactions incubated for 10, 20 or 30 min at 45 °C prior to the addition of 300 µL of DNS. Control reactions (blanks that determine the presence of reducing sugars in the starting mixture) contained all the same reagents except that DNS was added prior to the addition of enzyme sample. To determine the amount of reducing sugar produced during the enzyme catalyzed reaction the ABS^540nm^ of the control was subtracted from the enzyme reaction and resulting net gain in ABS^540nm^ converted into enzyme units µmol/min/µg. protein.

Cellobiohydrolase and β-glucosidase were assayed using *p*NPC, *p*-nitrophenyl β-d-cellobioside or *p*-nitrophenyl β-d-glucoside (pNPG) (Sigma Aldrich, St. Louis MO)) as a substrate, respectively and activity measured by the release of *p*-nitrophenyl that absorbs at ABS^420nm^ on a TECAN microwell reader. Briefly to 570 µl of 4 mM *p*NPC, 50 mM ammonium acetate buffer 5–10 µl of total extracellular protein extract (treated as described in 2.2) was added and reactions incubated for 5, 10 or 30 min at 45 °C prior to the addition of 60 µl of 2 M sodium carbonate. Control reactions contained all the same reagents except that 2 M sodium carbonate was added prior to the addition of enzyme sample. To determine the amount of *p*-nitrophenyl produced during the enzyme catalyzed reaction the ABS^420nm^ of the control was subtracted from the enzyme reaction and resulting net gain in ABS^420nm^ converted into enzyme units µmol/min/µg protein.

### Production of xylanases and cellulases with PPTB

Fermentation experiments examining the here constructed strains, PFIX7, PFIX7-EA and PFIX7-BA using PPTB were done in shaker flasks. The concentrated pre-hydrolysate was adjusted with water to a 30 g/l xylose-concentration and amended with mineral salts as described in Clutterbuck [59]. The inoculum was 1 × 10^5^ spores/ml medium and fermentations were carried out at 37 °C in an orbital shaker at 120 rpm for 72 h. Samples were taken and the supernatants stored at − 20 °C for later analysis. All experiments were done in triplicates.

### Determination of the phenolic content and sugar concentrations

The total phenolic content was analyzed according to the Folin–Ciocalteau method [74]. Briefly, properly diluted samples (200 µl) were added to distilled water (800 µl) and mixed with Folin–Ciocalteau regent (500 µl). Sodium carbonate (2.5 ml, 20% w/v) was added after 3 min and the samples were incubated in the dark for 30 min. The absorbance was measured at 725 nm using a photometer. Vanillin was used as external standard.

The concentrations of glucose, xylose, arabinose, acetic acid, furfural and 5-HMF in the pre-hydrolysate and cultivation samples were determined by HPLC measurements (Agilent 1200 Series). The HPLC was equipped with a pump unit, an autosampler unit, a refractive index detector unit and a computer software-based integration system (LC ChemStation). The MetaCarb 87H column was maintained at 80 °C at the flow rate of 0.5 ml/min with 0.05 M H_2_SO_4_ as the mobile phase. Peaks detected by refractive index were identified and quantified by comparison with the retention times of authentic standards.

## Supplementary information

**Additional file 1.** Construction of hemicellulose induced cellulase production *Aspergillus nidulans* strains.

## Data Availability

All data generated or analyzed during this study are included in this published article and its Additional file 1.

## Abbreviations

5-HMF: 5-hydroxymethylfurfural
ABS: absorbance
BglA: β-glucosidase
C6-sugars: glucose containing liquors
C5-sugars: pentose containing liquors
CbhB and CbhC: cellobiohydrolase B and C
CMC: carboxymethylcellulose
DNS: dinitro salicylic acid
EglA and EglB: endoglucanase A and B
GAT: gibson assembly technology
*gpdAp*: G3P dehydrogenase promoter
HPLC: high performance liquid chromatography
LCB: lignocellulosic biomass
LC–MS/MS: liquid chromatography-tandem mass spectrometry
PASC: phosphoric acid swollen cellulose
PCR: polymerase chain reaction
*p*NPC: *p*-nitrophenyl β-d-cellobioside
*p*NPG: *p*-nitrophenyl β-d-glucoside
PPTB: pentosan containing pre-treated biomass liquor
SDS: sodium dodecyl sulfate
SDS-PAGE: SDS polyacrylamide gel electrophoresis
WT: wild type A773 strain
XynC: xylanase C
*xynCp*: xylanase C promoter
XlnR: binuclear zinc finger transcription factor
