# Supplementary material for: Redesigning the Aspergillus nidulans xylanase regulatory pathway to enhance cellulase production with xylose as the carbon and inducer source
Source: Microb Cell Fact. 2019 Nov 7;18:193. doi: 10.1186/s12934-019-1243-5 (PMC6839167; doi:10.1186/s12934-019-1243-5)

**ADDITIONAL MATERIAL**

**Title:** Construction of hemicellulose induced cellulase production *Aspergillus nidulans* strains

**Authors:** Patrick Ballmann1, Jorge Lightfoot2, Michael Müller1 Stephan Droege1 and Rolf Prade2*

**Affiliations:** 1- Prüf- und Forschungsinstitut Pirmasens e.V., Marie-Curie-Strasse 19, 66953 Pirmasens, Germany

2- Department of Microbiology & Molecular Genetics, Oklahoma State University, Stillwater OK USA

**Contact email:** prade@okstate.edu

**Keywords:** *Aspergillus nidulans,* xylose induced cellulase production, cellulose hydrolysis, cellulases, lignocellulosic biomass pretreatment

**Abstract:** Here we describe how we reengineered *Aspergillus nidulans* cellulase promoters so that cellulase production is induced by xylose. Placing a xylanase promoter in front of a cellulase gene model was not enough to drive high levels of cellulase production. We than constructed a xlnR constitutive overexpressing strain and introduced *xynp*::*cellulase* constructs to achieve hyper production of cellulases induced by xylose.

**Method details**

Three types of strains were constructed in this study; **First** the resident CbhC (AN0494) promoter (*cbhCp*) was replaced with four xylanase promoters (*xynABCEp*) in such a way that recombinant strains induce the production of cellobiohydrolase by xylose, **second** a XlnR_(ORF)_ overexpression strain (PFIX7) was constructed by *pabaA* ectopic integration of a *gpdAp*::*xlnR_(ORF)_* DNA fragment, and **third**, xylose induced client protein constructs were randomly introduced into a XlnR overexpressing strain (PFIX7).

In all types of strain constructions, a linear hybrid recombinant DNA fragment was synthesized using Gibson Assembly Technology, GAT (Gibson, 2011; Gibson, 2014) using hybrid primers, Gibson Assembly Master Mix (New England Biolabs, US) and Phusion DNA Polymerase (New England Biolabs, US). DNA fragment size and DNA sequence verified hybrid DNA fragments were transformed into A773 or PFIX7 protoplasts. In the case of promoter replacements, a single gene replacement event at the cbh1 locus was selected for each *xyn(p)* promoter replacement by uracil/uridine sufficiency and by diagnostic PCR showing single integration (replacement) into the *cbhC* locus. For the XlnR overexpression the hybrid DNA fragment was integrated into the pabaA locus by a double crossover event disrupting it. Recombinants with a single gene replacement event were searched with diagnostic PCR and the resulting strain PFIX7 tested for XlnR over-expression.

For the client protein xylose induced strains we created plasmids carrying the *pUC18UP*::*pyroA*: *xynCp*::C**LIENT_ORF_**::*pUC18DWN* GAT construct that was transformed into PFI-X7 (XlnR overexpressing) strain and recombinants selected based on the level of client protein production rates. Even though we did not check for multiple integration events in single transformants we screened at least 100 transformants for high secretion levels of client proteins.

**A Construction of hemicellulose induced cellobiohydrolase production strains.**


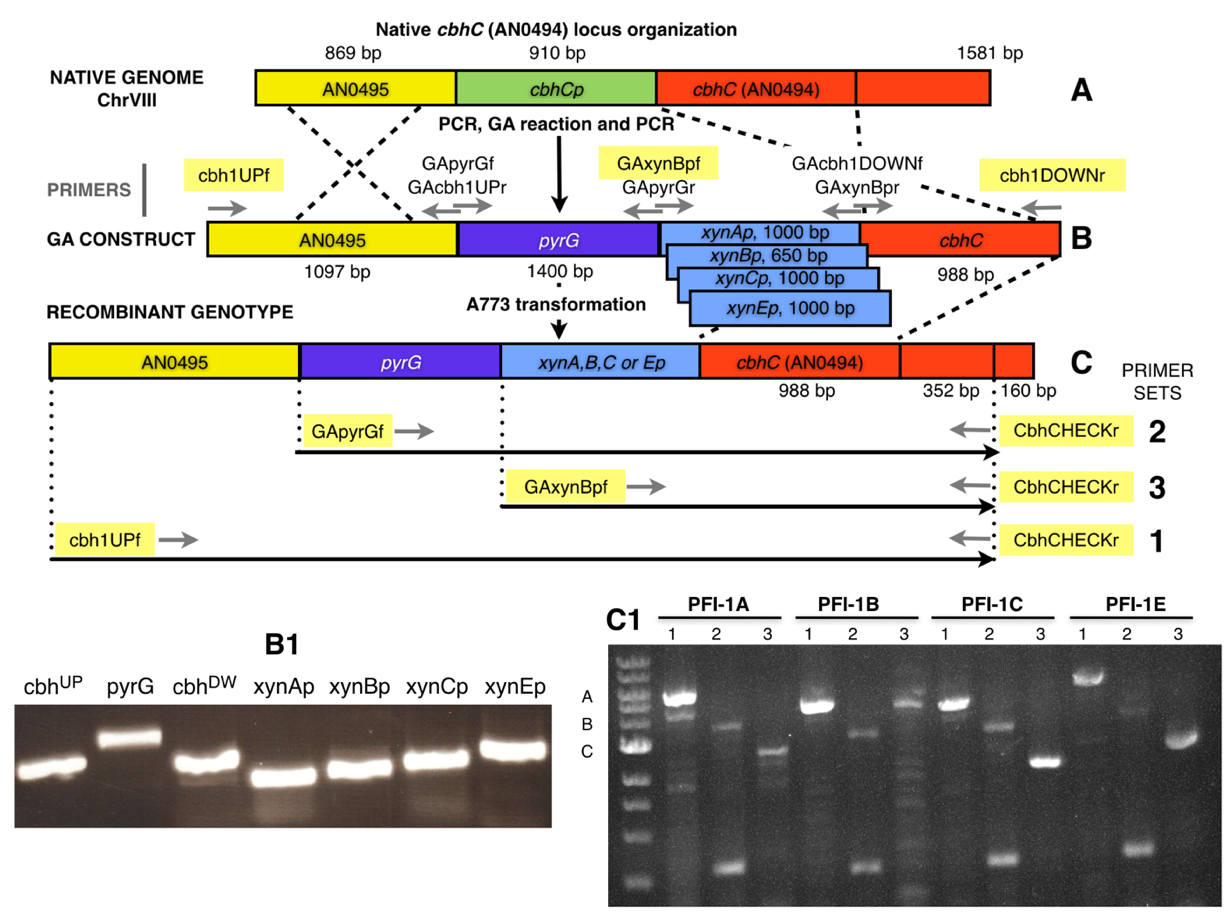


**Figure S1.** Construction of xylose induced cellobiohydrolase production strains.

*cbhC* promoter engineering outline. A. Native *cbhC* (AN0494) locus organization on chromosome VIII. **B** and **B1**. Gibson Assembly construct (GA). PCR generated DNA fragments, *cbh^UP^* (AN0495), *pyrG* (selectable marker) four *xyn* promoters (see **Table S1** for gene data), *xynAp*, *xynBp*, *xynCp* and *xynEp*, and *cbh^DW^* (AN0494). PCR fragments were amplified from *A. nidulans* FGSC4 (wild-type) genomic DNA template and GA hybrid primers (see **Table S3**). DNA fragments with hybrid ends (shown in **B** and **B1**) were amalgamated together using Gibson Assembly Master Mix (Gibson, 2011; Gibson, 2014). **C** and **C1**. The GA linear hybrid recombinant DNA segment (**B**) was transformed into A773 protoplasts and a single gene replacement (double crossover) event that describe the recombinant genotype shown in **C** was screened by diagnostic PCR. **C1** shows PCR products generated from genomic DNA of putative transformants, PFI-1A, PFI-1B, PFI-1C and PFI-1E with primer sets **1**, **2** and **3** identifying bands **A**, **B** and **C**, respectively (**C1**).

**B Construction of constitutive XlnR over expression strains.**


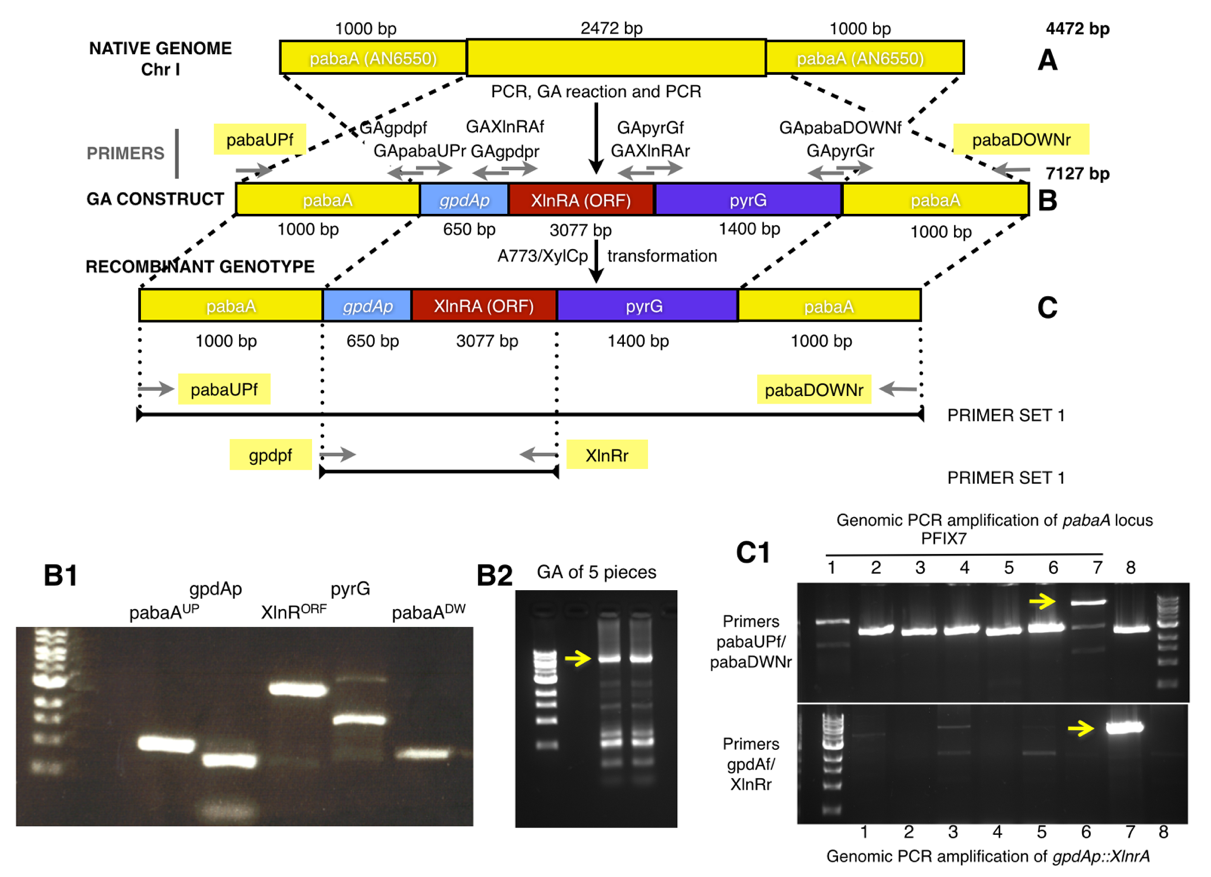


**Figure S2** Construction of XLNR overexpression strains.

XlnR transcription factor engineering outline. **A**. Native *pabaA* (AN6550) locus organization on chromosome I. **B**. Proposed GA construct. Five PCR generated DNA fragments, *pabaA^UP^*, *gpdp*, *xlnR^ORF^*, *pyrG*, *pabaA^DW^* (see **Table S1** for gene data). PCR fragments were amplified from *A. nidulans* FGSC4 (wild-type) genomic DNA template and GA hybrid primers (**Table S3**). DNA fragments with hybrid ends (shown in **B1**) were amalgamated together using Gibson Assembly Master Mix (Gibson, 2011; Gibson, 2014), and the 7 kb fused GA DNA fragment amplified with Phusion DNA polymerase (**B2**).

**C** and **C1**. The GA linear hybrid recombinant DNA segment (**B2**) was transformed into A773 protoplasts and a single gene replacement (double crossover) event at the *pabaA* locus was selected among transformants with *pabaA* auxotrophy and *pyrG* sufficiency. Further transformants were screened by diagnostic PCR and **C1** shows PCR products generated from genomic DNA of putative transformants. PFIX7 shows a *pabaA* disruption (in **C1**, primers pabaUPf/pabaDWr and *gpdp*::*xlnRA* fusion, in **C1**, primers gpdAf/XlnRr).

**C Construction of xylose induced overproduction of client (cellulase) proteins.**

**
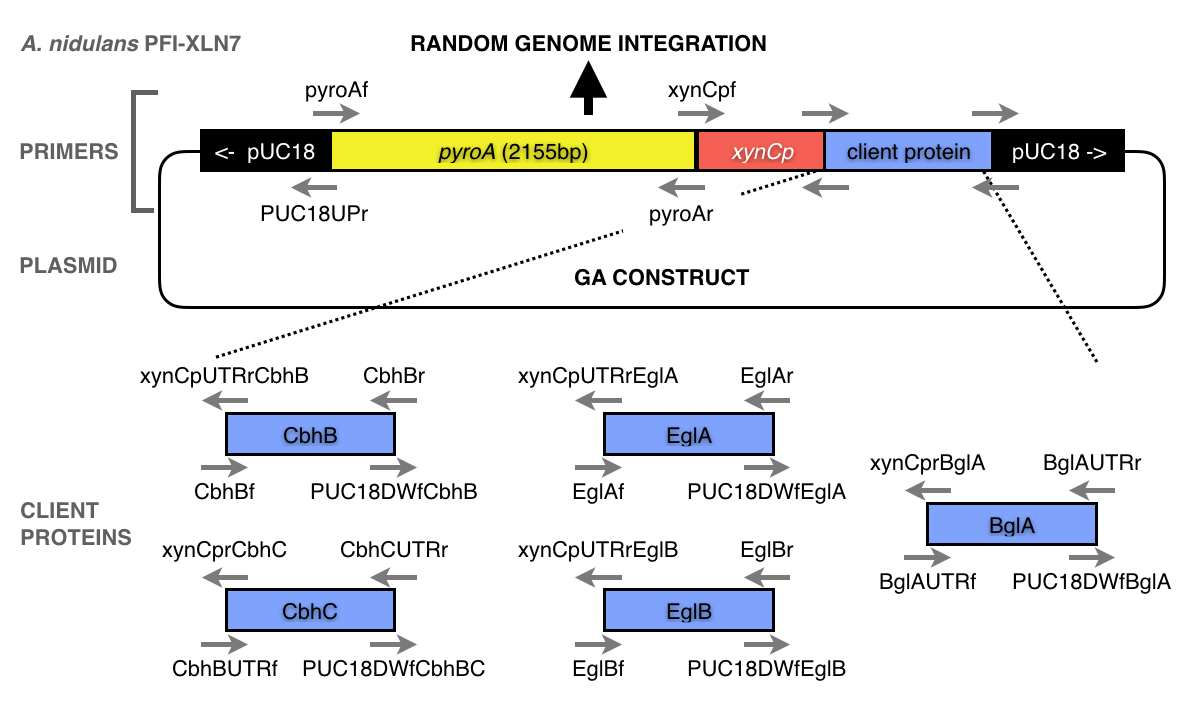
**

**Figure S3** Construction of strains that over secrete client proteins using xylose, hemicellulose or PPTB as a carbon source.

pUC18 based plasmids were constructed using common DNA fragments (*pyroA* and *xynCp*) fused to various client protein ORFs (mRNA), CbhB, CbhC, EglA, EglB and BglA (see **Table S1** for ORF/mRNA definitions). All constructs were done using GA technology with hybrid primer sets (see **Table S3**) PCR amplifying the pUC18 plasmid from pUC18 template DNA and other hybrid primer sets (see **Table S3**) PCR amplifying *pyroA*, *xynCp* and client protein ORFs (see **Table S1**) from *A. nidulans* FGSC4 genomic DNA. DNA fragments were fused with Gibson Assembly Master Mix (Gibson, 2011; Gibson, 2014), and plasmids recovered by transformation into DH5 ultracompetent *E. coli* cells.

Positive plasmids were directly transformed into PFIX7 protoplasts and recombinants selected based on complementation of pyridoxine (*pyroA*) requirement. Because multiple integrations could enhance the production of individual client proteins, 100 transformants for each client protein construct were selected based on their ability to secrete and accumulate the specified client proteins. The best preforming strains selected were PFIX7-BA, PFIX7-EA, PFIX7-EB, PFIX7-CB and PFIX7-CC (see **Table S2**).

**References**

Gibson, D.G. 2011. Enzymatic assembly of overlapping DNA fragments. Methods Enzymol, 498, 349-61.

Gibson, D.G. 2014. Programming biological operating systems: genome design, assembly and activation. Nat Methods, 11(5), 521-6.


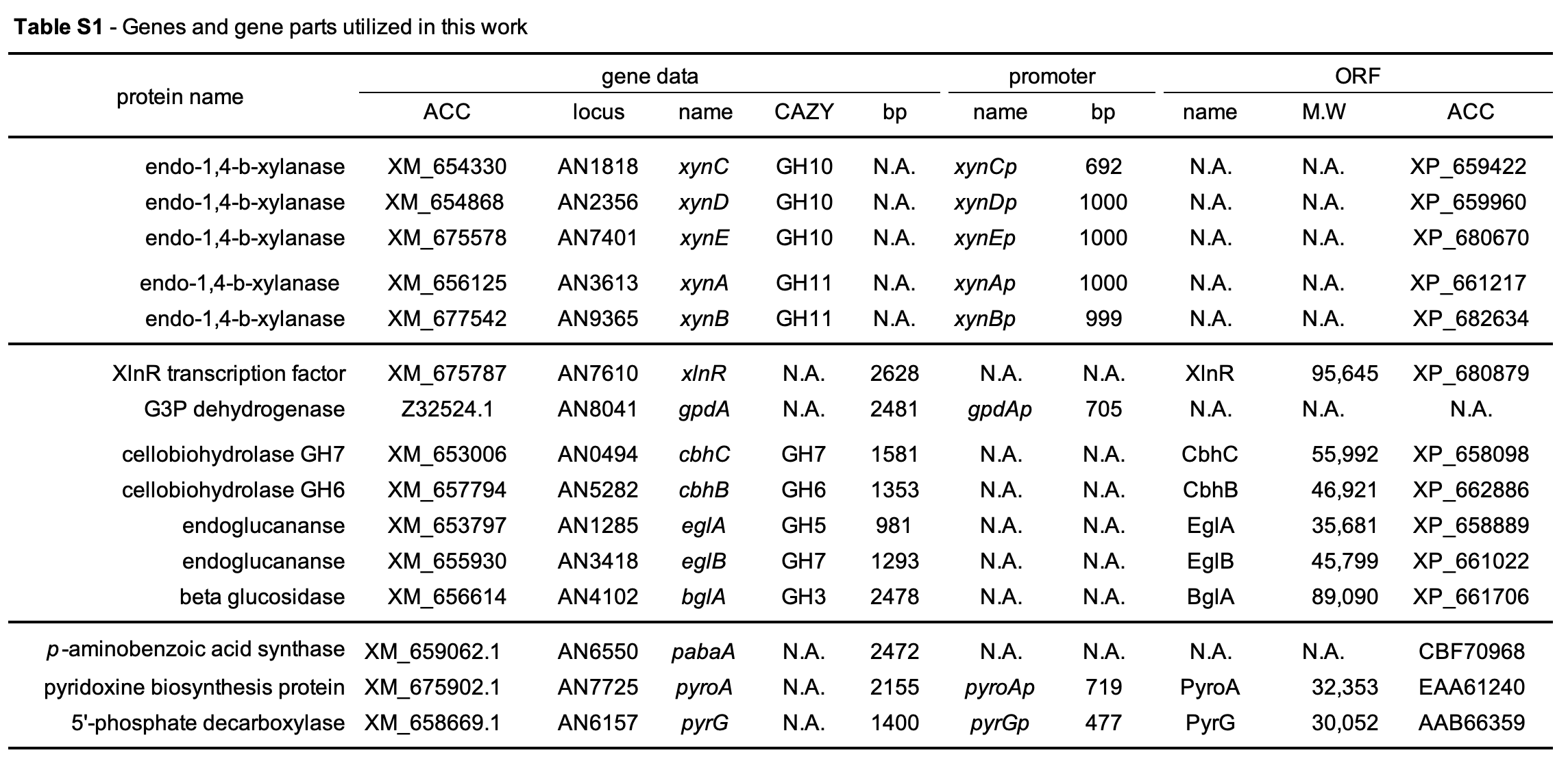


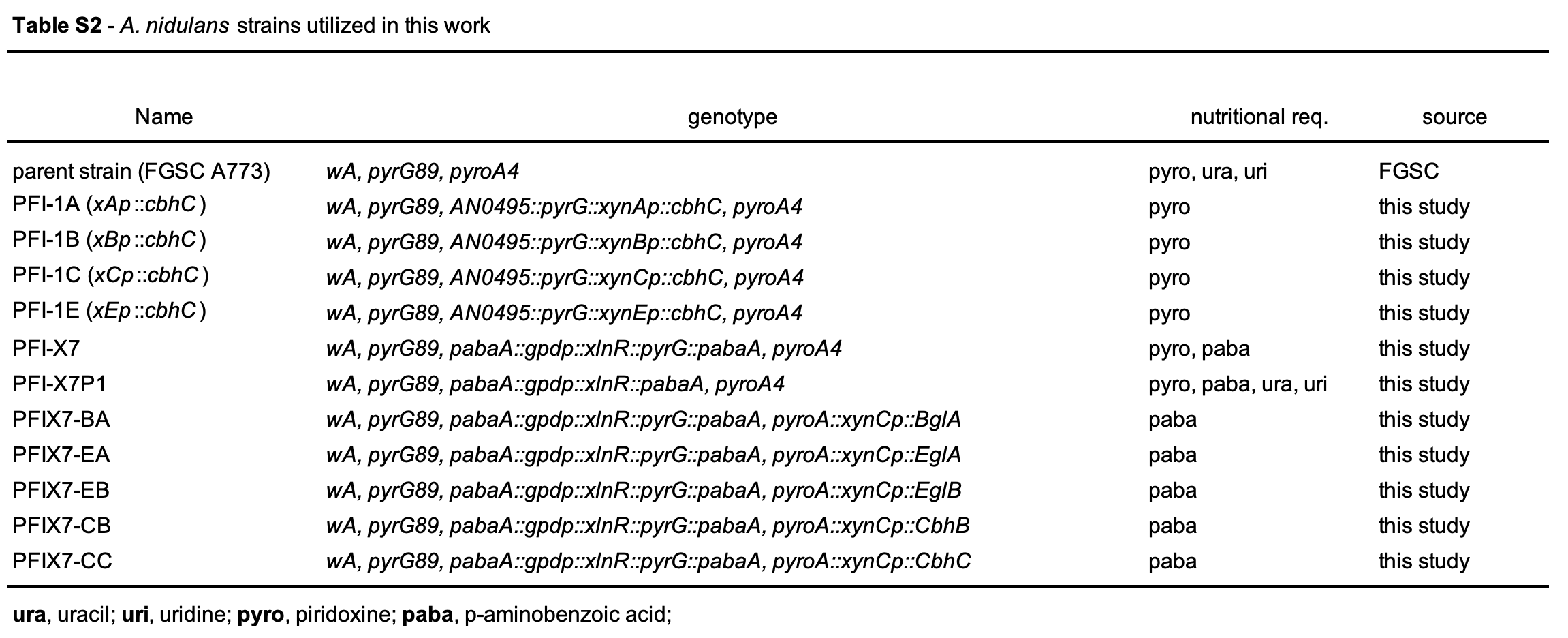


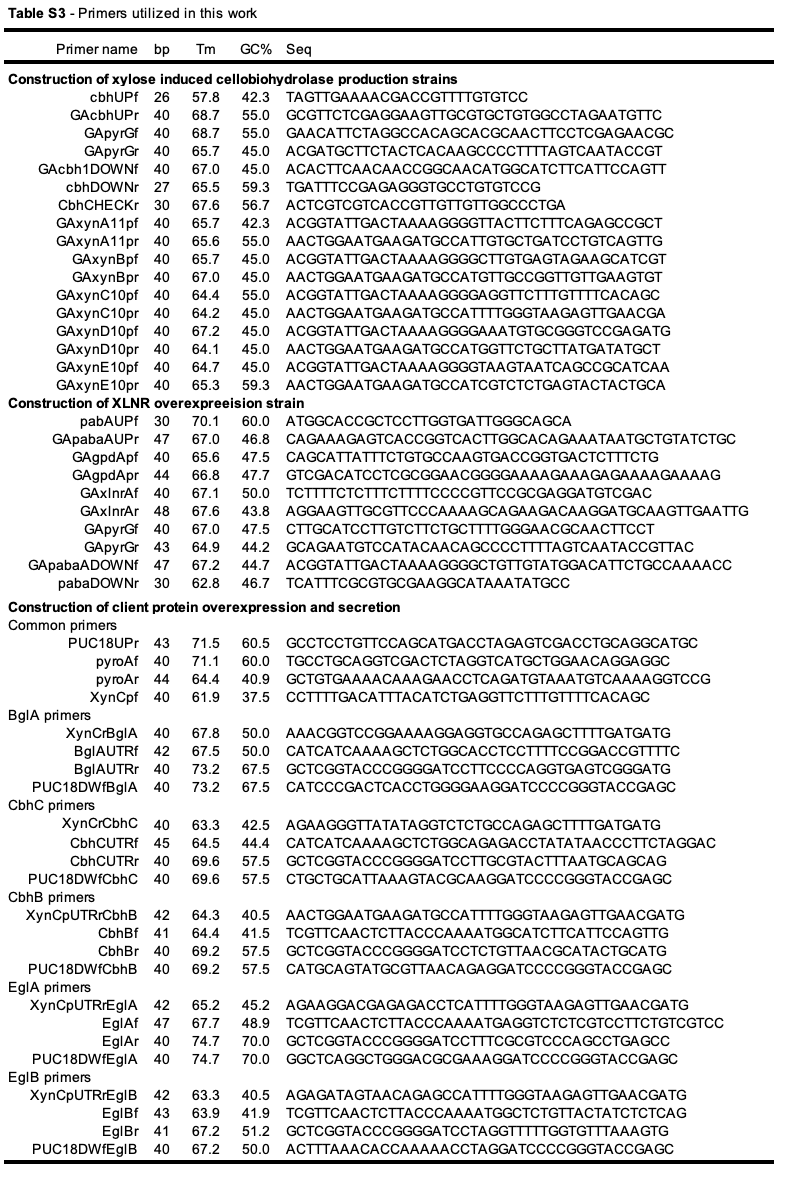

Supplement: Supplementary file 1 — Additional file 1. Construction of hemicellulose induced cellulase production Aspergillus nidulans strains. [file 12934_2019_1243_MOESM1_ESM.docx]
